# Spontaneous Retroperitoneal Hemorrhage (Wunderlich Syndrome) due to Large Upper Pole Renal Angiomyolipoma: Does Robotic-Assisted Laparoscopic Partial Nephrectomy Have a Role in Primary Treatment?

**DOI:** 10.1155/2013/498694

**Published:** 2013-09-11

**Authors:** Achilles Ploumidis, Ioannis Katafigiotis, Maria Thanou, Nikos Bodozoglou, Labros Athanasiou, Antonios Ploumidis

**Affiliations:** ^1^Department of Urology, Athens Medical Center, 15125 Athens, Greece; ^2^Department of Urology, Laiko General Hospital, University of Athens, 18535 Athens, Greece

## Abstract

Spontaneous rapture with consequent retroperitoneal hemorrhage (Wunderlich's syndrome) is the complication mostly feared from large renal angiomyolipomas (RAMLs). In hemodynamic stable patients, minimal invasive therapies have superseded open surgery as the mainstay of treatment, with contemporary cases mostly treated by selective arterial embolization. Robotic-assisted laparoscopic partial nephrectomy (RALPN) is an established minimal access treatment that has been used in the past for benign and malignant lesions of the kidney in the elective setting, but rarely in urgent situations as primary treatment. We present a case of a ruptured RAML in a young female treated effectively by RALPN.

## 1. Introduction

Renal angiomyolipomas (RAMLs) constitute a rare benign lesion with an incidence that fluctuates between 0.1% and 0.22% [1, 2]. Although benign, their rich neovascularization has a tendency for spontaneous rupture with consequent hemorrhage, which in some cases can be life threatening. This complication is proportional to size of the tumor, the grade of angiogenic component of the tumor, and the presence of tuberous sclerosis [3]. Furthermore, Wunderlich's syndrome is an emergency medical condition that refers to a spontaneous nontraumatic bleeding confined to the perinephric space caused by various etiologies but most frequently by renal angiomyolipoma [4]. It is a potential fatal complication of RAML and must be treated promptly and aggressively [5]. Although robotic-assisted laparoscopic partial nephrectomy has been implemented as an elective treatment for benign and malignant lesions of the kidney, it has rarely been used in urgent situations as primary treatment. We propose robotic-assisted laparoscopic partial nephrectomy (RALPN) as not only a feasible but also a safe alternative for the treatment of a bleeding angiomyolipoma by presenting a case of a ruptured RAML in a young female treated effectively by this modality. 

## 2. Case Presentation 

**Figure 1:**
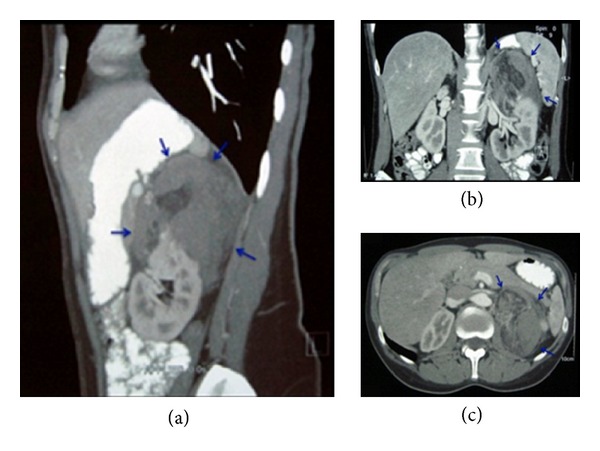
CT imaging of the abdomen depicting AML of the left kidney. (a) Sagittal plane. (b) Coronal plane. (c) Transverse plane.

A 32-year-old female was admitted to the emergency department with a sudden left-sided flank pain. The medical history and clinical examination was inconclusive, while the abdominal ultrasound revealed a left renal mass associated with retroperitoneal hemorrhage. The patient was hemodynamically stable and underwent immediate abdominal CT (Figure 1) that demonstrated a ruptured 7.6 cm RAML of the left upper pole with a large retroperitoneal haematoma. The patient under active monitoring was managed conservatively for 12 hours, during which a second abdominal CT revealed no active hemorrhage. Definitive treatment was decided by robotic-assisted renal sparing surgery, with the intention of enucleating the AML and removing of the hematoma. 

**Figure 2:**
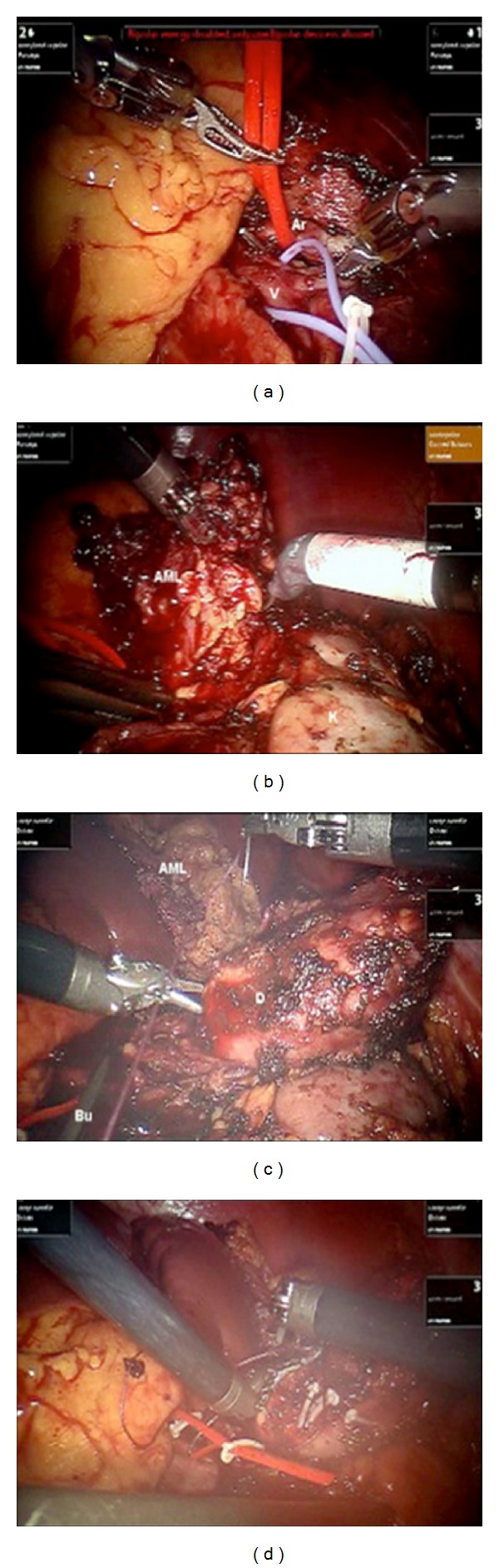
Intraoperative images during RALPN. (a) The renal artery (Ar) and vein (V) are identified. (b) Dissection of the AML from the upper pole of the kidney (K). (c) Clamping the renal artery with a laparoscopic Bulldog clamp (Bu) for suturing the renal defect (D). (d) Suturing the renal defect.

After induction of general anesthesia and endotracheal intubation, a nasogastric tube and a bladder catheter were inserted. The patient was placed and safely padded in a 45-degree modified right decubitus position, and the table was flexed near the posterior iliac crest. A 3-arm transperitoneal robotic approach was used with a 0-degree lens. The camera port was placed on the navel, while the two robotic ports were placed 10 cm from the camera port with the left robotic port placed in the midline and the right lateral and level with the navel. Two assisting ports were placed between the camera and robotic ports. Intraoperatively, the left colon was reflected from the posterior peritoneum, and the renal artery and vein were individually dissected, while a vessel loop was used for identification (Figure 2(a)). Dissection of the RAML was facilitated by mobilizing the kidney from its attachments (Figure 2(b)). When dissection was no longer possible without ischemia, a laparoscopic bulldog clamp (Aesculap Surgical Technologies, USA) was placed in the renal artery (Figures 2(b) and 2(c)). The renal defect was sutured with a sliding clip renorrhaphy by interposing Hem-o-lockTM clips (Weck Closure Systems, USA) to compress the renal parenchyma (Figure 2(d)). The clamp was removed and the surgical field checked for hemorrhage. The specimen was placed in a laparoscopic entrapment bag and removed from the umbilical port. A drain was placed through the incision of the right robotic trocar. 

**Figure 3:**
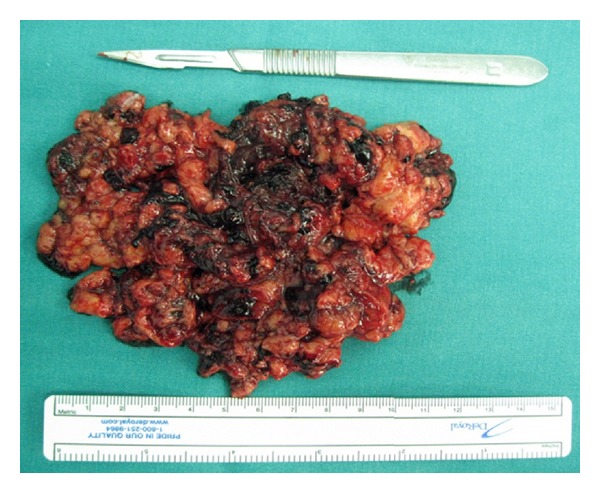
Macroscopic appearance of the ruptured AML.

Intraoperatively blood loss was 360 cc, which reflected the intense vascularization of the AML. Operative time was 210 min, while the warm ischemia time did not exceed 20 min. No intraoperative complications were encountered, and the postoperative period was uneventful. The drain and Foley catheter were removed, the first and third days, respectively, while the patient was discharged the third day. The pathology result was consistent with AML with no sign of malignancy (Figure 3). The patient was followed with abdominal CT and blood examinations, and one year after surgery no residual disease was noticed and the renal function remained normal. A written consent was obtained from the patient in order to present the case. 

## 3. Discussion 

 Wunderlich's syndrome is a life-threatening complication of RAML that can occur in up to 50% of patients with tumors larger than 40 mm, while 33% of the patients with bleeding RAMLs can develop hypovolemic shock [5, 6]. Different options for the management of a ruptured RAML have been applied both with the use of open surgery and minimal invasive techniques. Conventional management of a symptomatic AML is achieved with the open surgery either by the enucleation of the AML or by partial or radical nephrectomy [6]. Minimal invasive techniques have also been used for the treatment of asymptomatic RAML and laparoscopic, RALPN, selective renal arterial embolization (SAE), radiofrequency ablation, and cryoablation constitute the increasingly applied minimal invasive options [6–12]. Considering the fact that the patient was very young our goals were firstly to treat the ruptured AML readily with safety without compromising the life of the patient and secondly to try to preserve the kidney. Having the opportunity to apply all the suggested from the literature options for the treatment of a ruptured RAML, RALPN was the treatment of choice which was performed from a highly experienced A.P. with the use of the robot surgeon to combine the benefits both of a minimal invasive technique and of the preservation of the kidney. SAE has been proposed both as definitive treatment or as a useful component of a definitive treatment for the RAML with the preoperative embolization to constitute a recommended way to avoid excess blood loss during surgery [6, 9, 10, 13]. Although SAE can prevent renal loss and spare surgery and in long term can preserve the majority according to each case of the renal parenchyma, we rejected SAE as a definite treatment due to the fact that recurrence and reembolization can occur; surgery is not always avoided; a definite pathological diagnosis is not achieved as in surgery adding more stress to the patient; and mainly a more precise lifelong surveillance is needed even in a successful SAE for a RAML, not easily accepted and followed from a young patient [6, 14–16]. We did not perform a preoperative embolization since the patient was hemodynamically stable and there was no need for a further delay, and of course the reduction of the tumor volume can be only modest in a such short time between the two interventions [17]. To our knowledge, there is only one reference to the literature suggesting RALPN as a definite treatment of a ruptured RAML supporting our choice in this case. The benign nature of the RAML renders partial nephrectomy the ideal way of treatment especially in a young patient such as in our case whose renal function remained normal both immediately postoperatively and one year after the surgery [14]. We believe that RALPN can be applied not only in a scheduled surgery but in experienced hands can also with safety treat a ruptured RAML offering the established benefits of the robot both to the patient and the surgeon.
